# Comparing point counts, passive acoustic monitoring, citizen science and machine learning for bird species monitoring in the Mount Kenya ecosystem

**DOI:** 10.1098/rstb.2024.0057

**Published:** 2025-06-12

**Authors:** Ciira wa Maina, Peter Njoroge

**Affiliations:** ^1^ Centre for Data Science and Artificial Intelligence, Dedan Kimathi University of Technology, Nyeri, Kenya; ^2^ Ornithology, National Museums of Kenya, Nairobi, Kenya

**Keywords:** point counts, autonomous recording unit, indicator species

## Abstract

Biodiversity loss is a pressing challenge, with ecosystems across the world under threat from factors such as human encroachment, over exploitation and climate change. It is important to increase ecosystem monitoring efforts to provide actionable insights for ecosystem managers and to allow effective use of conservation resources. In this work, we compare traditional bird survey approaches using point counts with the use of autonomous recording units and citizen scientists’ data at two sites within the Mount Kenya ecosystem. We also present a new dataset of more than 20 h of recordings obtained from the Mount Kenya ecosystem and annotated by expert ornithologists, and investigate the use of large deep learning models to process these recordings. Our results are mixed, and at one site, autonomous recording units and traditional point counts yield similar conclusions when comparing relative abundance of species, while at the second site, conclusions differ. Our results indicate that citizen science is preferable to point counts and autonomous recording units in determining species lists for particular habitats. However, even with the use of multiple methods, our survey still misses rare species known to occur in the Mount Kenya ecosystem, indicating that even the use of multiple methods is not exhaustive.

This article is part of the theme issue ‘Acoustic monitoring for tropical ecology and conservation’.

## 1. Introduction

Ecosystem monitoring has always been an important aspect of conservation as it provides actionable insights for ecosystem managers and allows effective use of conservation resources [1]. The need for long-term ecosystem monitoring is now more pronounced since ecosystems across the world are under threat owing to factors such as human encroachment, overexploitation and climate change [2]. Traditional approaches to ecosystem monitoring include surveys of indicator species such as birds, bats and butterflies conducted periodically by trained experts [3]. These surveys remain the gold standard to assess ecosystem status but are unfortunately expensive and difficult to scale, limiting the ability to increase both their temporal and spatial sampling density [4].

To overcome these challenges, a number of approaches have been suggested, including the recruitment of citizen scientists, leveraging remote sensing and autonomous data collection using drones, camera traps and autonomous recording units (ARUs). Citizen science is promising as it allows large-scale data collection and also increases awareness of climate and ecosystem conservation. Citizen scientists have been successfully recruited to conduct bird surveys [5], map land use change [6] and even participate in the survey of threatened species [7]. Remote sensing is now an important tool in ecosystem monitoring and allows large-scale surveillance of land cover–land use change that could indicate habitat degradation [8].

Traditionally, ecosystem monitoring has involved the use of equipment such as cameras and audio recorders for data collection, often in the hands of trained experts. Recently, there has been a proliferation of low-cost devices for autonomous data collection of images, video and audio recordings from ecosystems of interest [9,10]. This has opened new opportunities to scale monitoring efforts, with the new bottleneck being the ability to analyse the large amounts of data generated. Fortunately, this increase in data collection and availability has been accompanied by advances in data analysis approaches, machine learning chief among them [11]. This has led to a great interest in developing machine learning approaches for environmental data processing. Several applications of machine learning to process data derived from camera traps, ARUs and remote sensing have been reported in the literature.

Machine learning applied to the analysis of camera trap or audio recording data has a long history, with early work applying techniques such as support vector machines to bird species detection [12]. Schneider *et al*. [13] describe early work in applying computer vision to species identification from camera trap data. In the past decade, deep learning has become the dominant machine learning approach with success in several domains, including image recognition, speech recognition and natural language processing [14–18]. Deep learning has been successfully applied to the recognition of species from camera trap images and audio recordings from ARUs such as the AudioMoth [19–21].

Data availability and readiness are critical for the successful application of deep learning to problems in species recognition. There have been several competitions that have led the way in making such data and the resulting models available for further research and deployment, including the Machine Learning for Signal Processing (MLSP) competition [22] and the annual BirdClef competitions [23]. Recently, several models have been made available based on state-of-the-art neural network architectures such as convolutional neural networks [24] and the transformer [25]. BirdNET and the recent Google Bird Vocalization Classifier (Perch) [26] (https://www.kaggle.com/models/google/bird-vocalization-classifier) are based on the EfficientNET architecture [27], an improved convolutional neural network architecture. The availability of these models allows researchers to use them to extract embeddings from local recordings to train models for local contexts and to use them to draw ecological conclusions.

A number of studies have leveraged these models to process novel datasets and have shown good results [28]. However, there is a need to explore the use of these models in the analysis of data collected for biodiversity monitoring. This is particularly important in the tropics, where most of the world’s biodiversity resides, but which is also under monitored. Machine learning has the potential to greatly improve coverage of biodiversity monitoring in these regions.

A number of studies have applied bioacoustics to the study of ecosystems by focusing on birds that serve as indicators of ecosystem health [29]. The application of bioacoustics to ecosystem monitoring involves the collection of audio recordings from the ecosystem of interest followed by either manual processing by ornithologists or machine learning‐based processing. Examples include studies focusing on individual species such as the Eurasian bittern [30], the Hawai‘i ‘ amakihi [31] and the European nightjar [32]. Other studies have sought to compare the use of acoustic recording with point counts (PCs) for bird species monitoring [33]. Studies focused on the African continent include projects in Kenya [34–36], Cameroon [37] and South Africa [38]. The conclusions of these comparisons are varied, with some reporting higher species richness results obtained by acoustics surveys [35] and others reporting the opposite [39].

In addition, there are efforts to use citizen scientists to monitor bird species in Africa, with the African Bird Atlas Project (ABAP) leading efforts to collect bird species distribution data using a robust and repeatable protocol [5]. The Kenya Bird Map (KBM) project is part of the ABAP and has collected bird species lists since 2012. These data are available at https://kenya.birdmap.africa/.

In this work, we aim to compare PCs, annotation of recordings from ARUs and observations from citizen scientists recorded on the KBM when used to monitor bird species in the Mount Kenya ecosystem. We focus on two sites, the Dedan Kimathi University Wildlife Conservancy (DeKUWC) and within the Mount Kenya National Park (MKNP), and are able to compare species richness estimates and relative abundance obtained from PCs, ARUs and KBM, as well as the effectiveness of different monitoring approaches in surveying forest birds. In addition, we demonstrate the application of modern machine learning methods to analyse audio recordings collected within the DeKUWC and MKNP. This work aims to illustrate the similarities and differences of PCs, ARUs and citizen science when applied to the Mount Kenya ecosystem and explore the use of state-of-the-art machine learning models to process ARU data from Mount Kenya. Use of machine learning models to process ARU data, coupled with knowledge on when we can reliably use acoustic surveys to monitor bird populations in place of traditional survey techniques such as PCs, will be useful in guiding future monitoring efforts. We also introduce a new dataset of audio recordings that can spur further research in machine learning applications for bird species monitoring [40].

## 2. Methods

### (a) Study area

**Figure 1.**
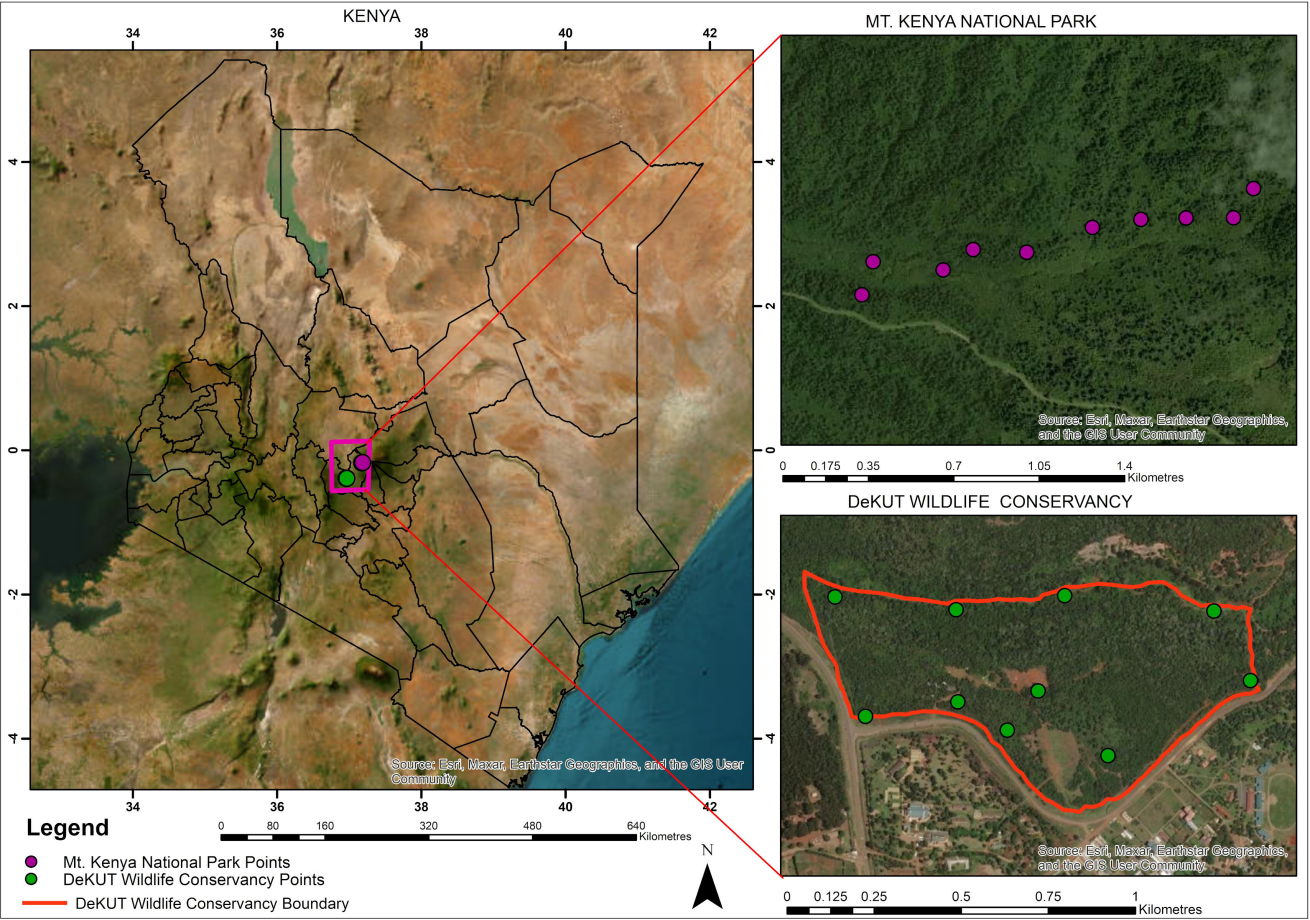
A map of the study area, showing Kenya and the two sites within the Mount Kenya ecosystem. Mount Kenya National Park (MKNP) is shown on the top right, while the Dedan Kimathi University Wildlife Conservancy (DeKUWC) is shown on the bottom right. In both cases, point count and co-located recorder locations are indicated.

The study was conducted in the Mount Kenya ecosystem at two sites, namely the DeKUWC and within the MKNP. The DeKUWC (0°23′17.0″S, 36°57′43.2″E) is a 120 acre conservancy located approximately 5 km from Nyeri town in central Kenya at an altitude of approximately 1800 m above sea level. The conservancy is managed by Dedan Kimathi University of Technology as a protected area with a number of wildlife species including impala, zebra, bushbuck, water buck, warthog and Sykes’ monkeys [41]. Vegetation at DeKUWC is dominated by indigenous forest with tree species such as the cape chestnut (*Calodendrum capense*), silver oak (*Brachylaena huillensis*), small fruited teclea, (*Teclea nobilis*) and arrow poison tree (*Arundinaria alpina*). As can be seen from figure 1 (bottom right panel), DeKUWC is close to human settlements.

Within the MKNP, data collection was performed near the Naro Moru gate (0°10′29.1828″S, 37°8′ 37.6944″E) on the western side of Mount Kenya at altitudes ranging from 2500 to 2700 m above sea level. MKNP is a protected area without human settlement, with an area of about 715 km${,}^{2}$ [42]. Common vegetation in the area includes mountain bamboo (*Arundinaria alpina*) and podo trees (*Podocarpus latifolius*). The area is also home to African elephants (*Loxodonta africana*) and Cape buffaloes (*Syncerus caffer caffer*).

Both the DeKUWC and the MKNP are within the African Highland biome and share common species [43]. They are also priority areas for conservation interventions [43].

### (b) Point count data

Fixed‐width PCs were conducted both at the DeKUWC and within the MKNP. Ten locations were sampled within each site, with locations *ca*200 m apart. These PC locations are indicated in figure 1. The PCs were conducted early in the morning between 06.30 and 11.30. At each PC location, the observer waited 1 min to allow a settling interval and then recorded all species observed within a radius of 25 m for the next 9 min. PC locations within the conservancy (figure 1, bottom right) were visited four times (40 PCs), while locations within the MKNP (figure 1, top right) were visited six times (60 PCs). Data collection was conducted between November and December 2017 and June and July 2018 in MKNP and between January and May 2017 in DeKUWC.

The PC data are analysed using species accumulation curve modelling to determine how close observed species numbers are to actual numbers present in the site [44]. In this model, the expected cumulative number of species S(t) is given by


(2.1) $$S(t)=\frac{a}{b}(1-\text{exp}(-bt)),$$

where t is a unit of effort (PC number in this case), a is a parameter that measures the rate of species increase at the beginning of the study and a/b is the asymptote. We compute the number of PCs required to reach 90% survey completeness as


(2.2) $$\begin{array}{lcr} & t_{0.9}=-\frac{1}{b}\ln\;\left(1-0.9\right). & \end{array}$$

### (c) Acoustic recordings

A previous study had obtained acoustic recordings from DeKUWC in 2016 using a Raspberry Pi-based recording system programmed to obtain a minute-long recording every 5 min [34]. Recordings, 2701 min long, sampled at 16 kHz, were obtained, with 300 of them annotated by ornithologists. The sampling frequency of 16 kHz was chosen because all species of interest vocalize below the Nyquist frequency of 8 kHz [45, ch. 3]. In this work, recordings were obtained at DeKUWC from the same 10 PC locations shown in figure 1 (bottom right) using an early version of the AudioMoth programmed to record for 1 min every 5 min between 6.00 and 11.00 [9,21]. The recorders were set up at the same time the PCs were conducted and operated for about two weeks. The sampling rate was 16 kHz, with samples stored at 16 bit resolution. Of these, 300 recordings were annotated by an ornithologist. Similarly, recordings were obtained at the same PC locations in the MKNP (figure 1, top right). Here, 900 recordings were annotated. Table 1 shows a summary of the data available [40].

**Table 1.** Audio data. DeKUWC, Dedan Kimathi University Wildlife Conservancy; MKNP, Mount Kenya National Park.

site	hours	availability
DeKUWC	5	https://doi.org/10.5061/dryad.d51c5b0c7
MKNP	15	https://doi.org/10.5061/dryad.d51c5b0c7

### (d) Kenya Bird Map data

The Kenya Bird Map data consist of species lists obtained within a geographical pentad, that is, a region spanning 5′ latitude and 5′ longitude (approximately 9 km by 9 km at the Equator; [5]). Observers visit all habitats within the pentad and record species in the order they are observed for a minimum of 2 h over a maximum of 5 days. This corresponds to the ‘full-protocol’, which is conducted by experienced birders capable of recognizing at least 90% of the species they encounter [5]. The ‘full-protocol’ is similar to timed species counts, which are useful in determining species lists in a region.

We analysed all the ‘full-protocol’ species lists from pentads within 20 km of our study sites collected over the same period as the PCs. This led to 12 full protocol species lists for MKNP and 46 for DeKUWC.

### (e) Statistical comparison of autonomous recording unit annotation, point counts and Kenya Bird Map data

One of the objectives of the study is to compare different survey methods for bird biodiversity monitoring. To compare PCs, annotations from ARUs and KBM data, we compare the frequency of occurrence of each species occurring at both DeKUWC and MKNP for the different methods. Each PC lasts 10 min while each audio recording is 1 min long. To simulate equal effort in order to make the PCs and audio annotations comparable in our statistical analysis, we randomly sample 10 recordings from each site and treat these as equivalent to a single PC and determine the species represented in the recordings. We simulate 40 such PCs for DeKUWC and 60 for MKNP to ensure equal effort between the PCs and audio recordings and determine the frequency of occurrence of each species. The frequency of occurrence for each bird species for the KBM data is determined by counting the number of lists in which the species appeared and dividing it by the number of lists per site.

We make pairwise comparisons for the three methods, and for each site we test for the normality of the differences between the frequency of occurrence for the two methods being compared for the species occurring at the site using the Kolmogorov–Smirnov test. The results of the test for normality are used to determine whether we use paired t-tests or the Wilcoxon signed-rank test to test the null hypothesis that the frequencies of occurrence for the species determined from PCs, ARU annotations or KBM data are drawn from the same distribution. If sufficient evidence exists to reject the null hypothesis, then we conclude that the two methods being compared differ. This approach was employed in [33].

### (f) Machine learning models

In this work, we test the performance of BirdNET [20] and Google’s Perch model [26] on the annotated audio recordings obtained at DeKUWC and MKNP to determine if these models can be used to help scale the use of ARUs within the Mount Kenya ecosystem. These models have been selected because they are publicly available, widely adopted within the bioacoustic community and have shown good performance in a number of studies [28,46,47]. Both BirdNET and Perch are trained to recognize Kenyan species, with approximately 62 and 93% of Kenyan species available in BirdNET and Perch, respectively. For each recording, we compare the species predicted by the models to the annotations. For BirdNET, the recordings are divided into 3 s segments, and species whose BirdNET score is greater than the default value of 0.1 in all of the segments are reported as predictions for the recording [48]. For Perch, the recording is divided into 5 s segments and the species corresponding to the maximum logit for each segment are reported as predictions for the recording if they correspond to species found in Kenya.

A number of species common in the Mount Kenya ecosystem are absent in the models, and we use Perch as a feature extractor to train bird classification models for the Mount Kenya ecosystem. The annotated audio recordings have multiple species, and we use the extracted embeddings to train a multi-label multi-layer perceptron classifier implemented in Python using Keras [49]. The output nodes correspond to the species to be classified and have sigmoid activations that output the probability of the presence of the species. The probability threshold used to determine species presence is selected to maximize accuracy on the training set by conducting a line search on the interval [0,1]. The loss function is binary cross entropy optimized using Adam [50]. 1280 dimensional embeddings are extracted from each of the twelve 5 s segments from the 1 min long recordings, and the mean of these embeddings is used as a feature for the recording. A similar approach was used in [28] to train models with as few as four recordings per species and showed good classification results.

Code to reproduce the experiments is available at [51] and the corresponding GitHub repository: https://github.com/DeKUT-DSAIL/ndege-zetu.

## 3. Results

### (a) Species richness

Overall, a total of 288 species were recorded during our study at both sites, with the number of species recorded by each method as follows: PCs (94), identification in audio recordings by expert ornithologists (100) and KBM data (243). A summary of species richness at each site identified via PCs, annotation of the audio recordings and from the KBM data is shown in table 2.

**Table 2.** Species richness at Mount Kenya National Park (MKNP) and the Dedan Kimathi University Wildlife Conservancy (DeKUWC). ARU, autonomous recording units; KBM, Kenya Bird Map; PC, point counts.

site(s)	survey method			
	point count	ARU	KBM	combined
DeKUWC	57	89	224	260
MKNP	48	35	125	144
both	94	100	243	288

**Figure 2.**
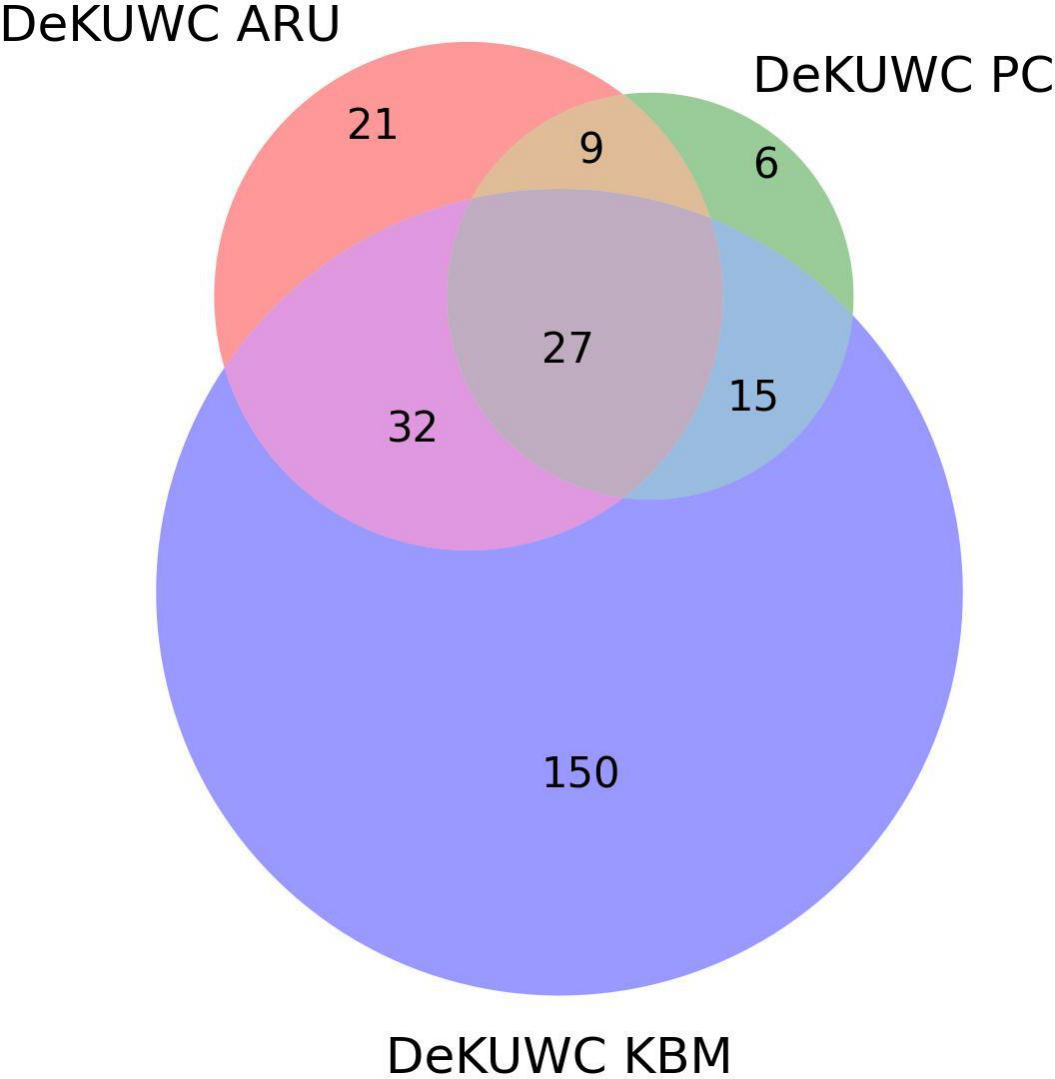
Comparison of species observed at the Dedan Kimathi University Wildlife Conservancy (DeKUWC). ARU, autonomous recording units; KBM, Kenya Bird Map; PC, point counts.

At DeKUWC, the largest number of unique species (150) was recorded from the KBM data, while the least number of unique species (six) was recorded from the PCs (figure 2). Of the 260 species recorded at DeKUWC, 177 were recorded by only one method while a total of 27 species were recorded by all the survey methods (figure 2).

**Figure 3.**
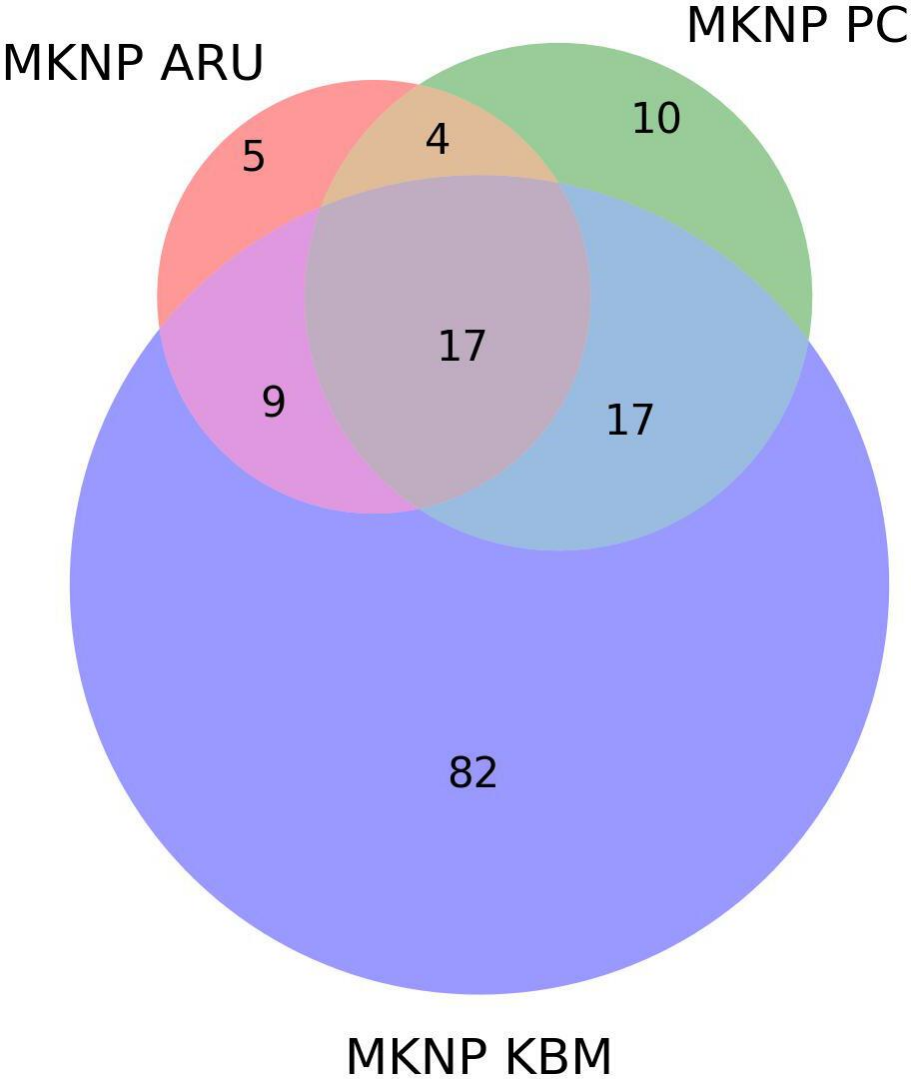
Comparison of species observed at Mount Kenya National Park (MKNP). ARU, autonomous recording units; KBM, Kenya Bird Map; PC, point counts.

**Figure 4.**
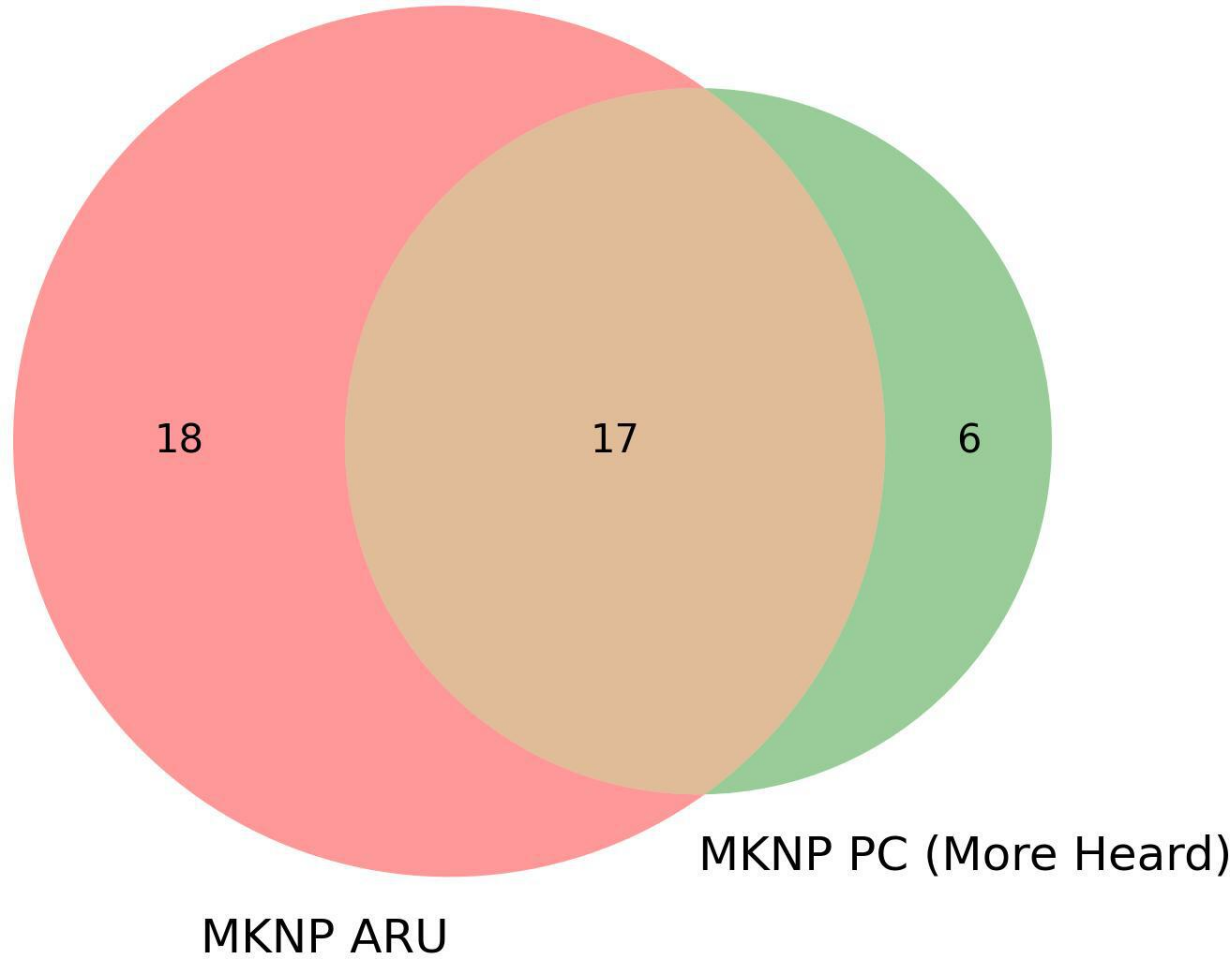
Overlap of species more seen than heard at Mount Kenya National Park (MNKP) and those observed in audio recordings. ARU, autonomous recording units; PC, point counts.

At MKNP, the largest number of unique species (82) was recorded from the KBM data, while the least number of species (five) was recorded from the ARU (figure 3). A total of 17 species were recorded by all the survey methods (figures 3 and 4).

**Figure 5.**
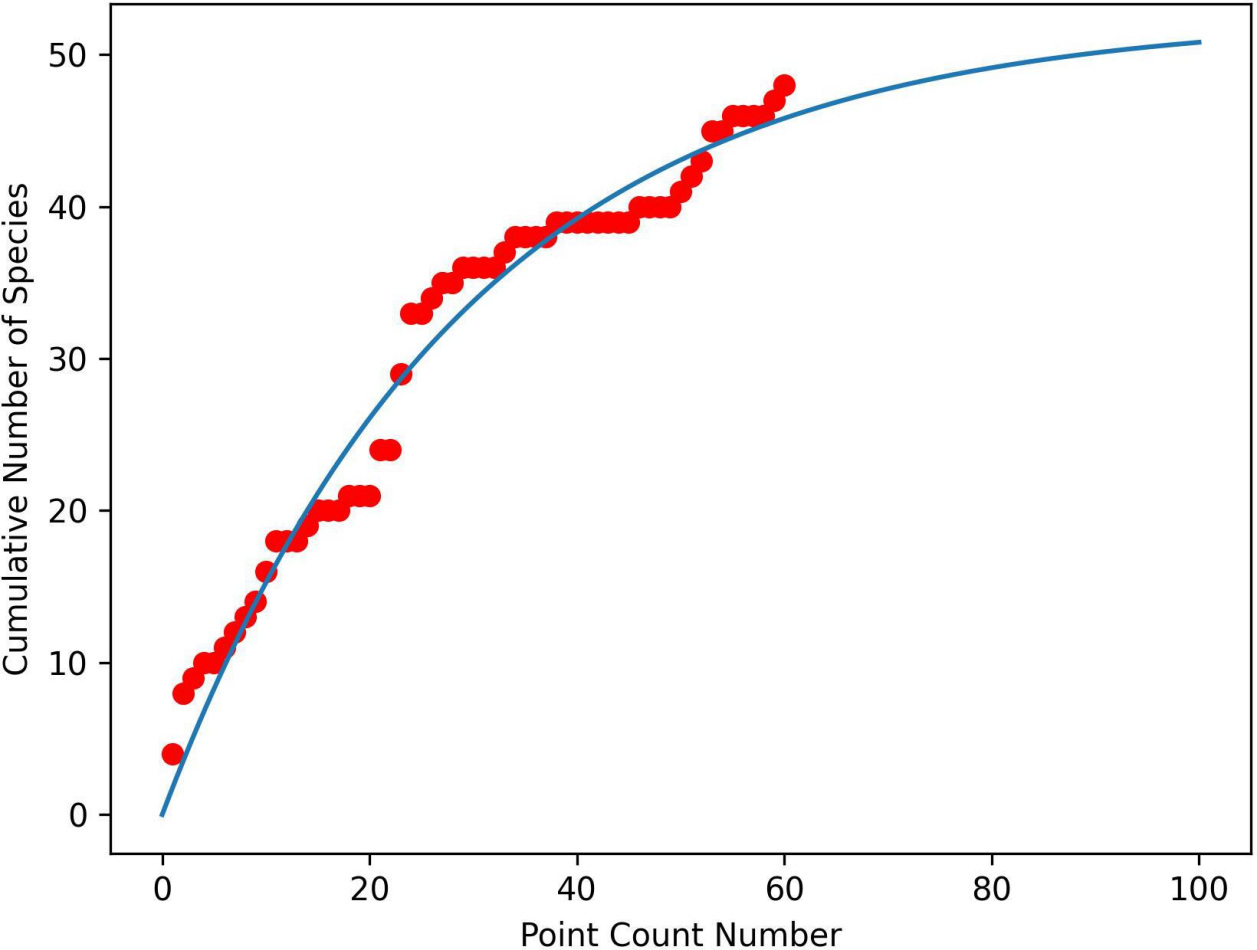
Species accumulation curve for Mount Kenya National Park. Dots represent the observations, and the line is the model fit given by equation (2.1).

**Figure 6.**
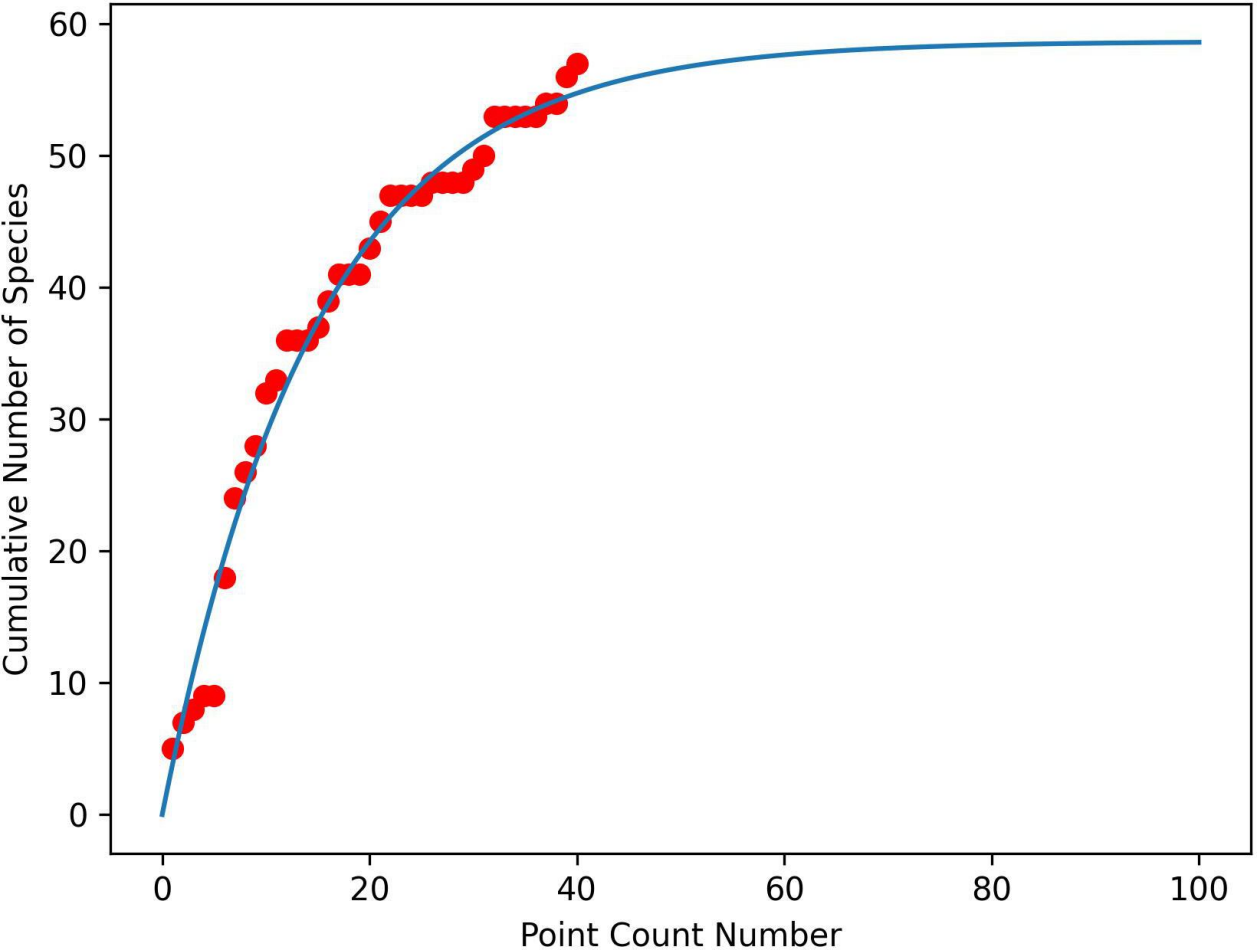
Species accumulation curve for the Dedan Kimathi University Wildlife Conservancy. Dots represent the observations and the line is the model fit given by equation (2.1).

The species accumulation curves are shown in figure 5 (MKNP) and figure 6 (DeKUWC). For DeKUWC, $t_{0.9}=34$, implying the 40 PCs were sufficient. The asymptote is approximately 59, close to the observed number of 57 (table 2). However, the larger numbers of species observed via ARU (89) and KBM (224) suggest that PCs may be unable to obtain an exhaustive list of species present at the site. For MKNP, $t_{0.9}=67$, slightly larger than the 60 PCs conducted, with an asymptote of approximately 53, which is also slightly larger than the 48 species observed.

### (b) Species composition

#### (i) Forest dependency

**Figure 7.**
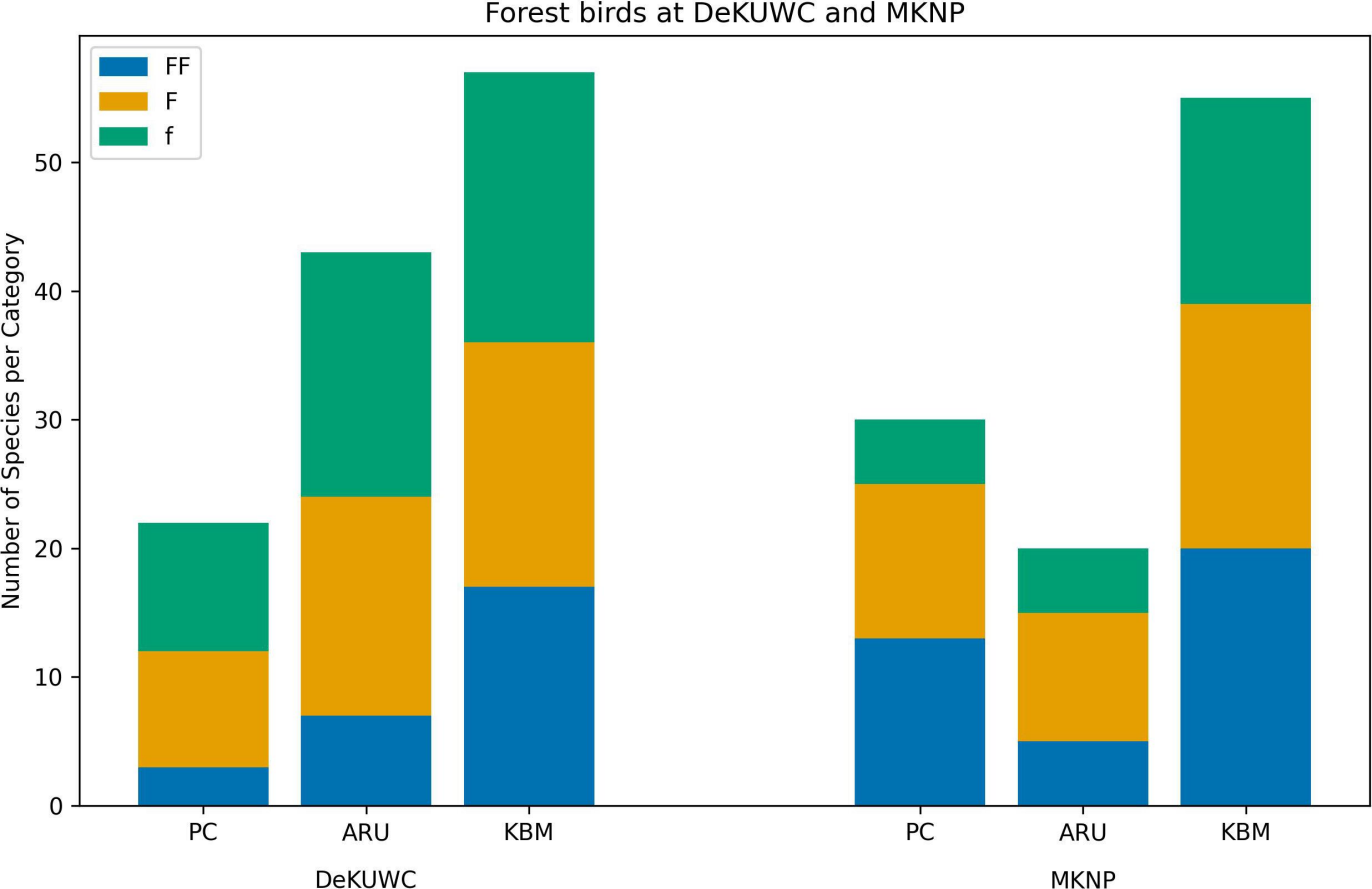
Number of forest birds at the Dedan Kimathi University Wildlife Conservancy (DeKUWC) and Mount Kenya National Park (MKNP). The species are classified as forest specialists (FF), forest generalists (F) or forest visitors (f) [52]. PC, point counts; ARU, autonomous recording units; KBM, Kenya Bird Map.

We examined the presence of forest specialist species captured using the three methods. Forest dependency is a measure of the relative condition of the forest [52]. Using the list of forest species from Kenya and Uganda and the classification of species as forest specialists (FF), forest generalists (F) or forest visitors (f) presented in [52], we classify the observed species as either FF, F or f. Most forest specialist species were captured by the KBM method at both sites, while at DeKUWC, the PC method captured the least number of forest specialist species (figure 7).

#### (ii) Species of conservation importance

Hinde’s babbler (*Turdoides hindei)* was the only globally threatened species recorded in the surveys (table 3). Two other species are listed as near-threatened (table 3; [53]). Hinde’s babbler was only recorded at DeKUWC and was captured in the KBM data as well as the ARU data. These three species are not very vocal.

**Table 3.** Species of conservation importance recorded at Mount Kenya National Park (MKNP) and the Dedan Kimathi University Wildlife Conservancy (DeKUWC) by point counts (PC), annotation of audio recordings (ARU) and citizen science (KBM).

species			site					
			MKNP			DeKUWC		
scientific name	common name	status	PC	ARU	KBM	PC	ARU	KBM
*Stephanoaetus coronatus*	crowned eagle	NT	✓	✓	✓	✗	✗	✓
*Buteo oreophilus*	mountain buzzard	NT	✓	✗	✓	✗	✗	✓
*Turdoides hindei*	Hinde’s babbler	VU	✗	✗	✗	✗	✓	✓

### (c) Detecting vocal species

For PCs conducted at MKNP, we recorded the cue (detection signal of whether heard or seen). We compared the number of species heard by the PC method to those recorded by the ARU. At MKNP, 23 out of the 48 species recorded were more often heard than seen during the PCs (table 4). Seventeen of these species (74%) were also recorded by the ARU (figure 4). The species not identified in the recordings are the African hill babbler, chin-spot batis, grey cuckooshrike, variable sunbird, white-bellied tit and yellow-crowned canary.

**Table 4.** Species more often heard than seen at Mount Kenya National Park..

scientific name	common name	number of observations	fraction heard
*Phylloscopus umbrovirens*	brown woodland warbler	50	0.94
*Bradypterus cinnamomeus*	cinnamon bracken warbler	38	0.95
*Apalis porphyrolaema*	chestnut-throated apalis	34	0.97
*Zosterops poliogastrus*	montane white-eye	25	0.84
*Pogonocichla stellata*	white-starred robin	21	0.86
*Poicephalus gulielmi*	red-fronted parrot	15	0.60
*Tauraco hartlaubi*	Hartlaub's turaco	13	0.92
*Chloropeta similis*	mountain yellow warbler	11	0.91
*Cyanomitra olivacea*	olive sunbird	8	0.50
*Stephanoaetus coronatus*	crowned eagle	6	1.00
*Parus albiventris*	white-bellied tit	4	0.75
*Muscicapa adusta*	African dusky flycatcher	3	1.00
*Pseudoalcippe abyssinica*	African hill babbler	3	0.67
*Pheoniculus bollei*	white-headed wood-hoopoe	3	0.67
*Andropadus nigriceps*	mountain greenbul	3	0.67
*Cinnyris venustus*	variable sunbird	1	1.00
*Coracina caesia*	grey cuckooshrike	1	1.00
*Accipiter tachiro*	African goshawk	1	1.00
*Apalis cinerea*	grey apalis	1	1.00
*Cinnyris mediocris*	eastern double-collared sunbird	1	1.00
*Pycnonotus barbatus*	common bulbul	1	1.00
*Batis molitor*	chin-spot batis	1	1.00
*Serinus flavivertex*	yellow-crowned canary	1	1.00

### (d) Frequency of occurrence (relative abundance)

The brown woodland warbler and chestnut-throated apalis were the most frequently recorded at MKNP by both PC and ARU methods (table 5), while the Hartlaub’s turaco was the most frequently recorded from the KBM data at the site. At DeKUWC, the grey-backed camaroptera and common bulbul were the most frequently recorded species by PCs, while the speckled mousebird, variable sunbird and white-eyed slaty flycatcher were the most frequently recorded from the KBM data (table 6). Two very vocal species, the grey-backed camaroptera and the yellow-whiskered greenbul, were the most frequently observed by ARUs.

**Table 5.** Frequency of occurrence for species identified at Mount Kenya National Park via point counts and annotation of audio recordings. PC, point counts; ARU, annotation of audio recordings, KBM, Kenya Bird Map.

species		survey method		
scientific name	common name	PC	ARU	KBM
*Phylloscopus umbrovirens*	brown woodland warbler	0.65	1.00	0.42
*Apalis porphyrolaema*	chestnut-throated apalis	0.53	0.87	0.67
*Zosterops poliogastrus*	montane white-eye	0.50	0.42	0.00
*Bradypterus cinnamomeus*	cinnamon bracken warbler	0.45	0.57	0.58
*Pogonocichla stellata*	white-starred robin	0.37	0.55	0.50
*Poicephalus gulielmi*	red-fronted parrot	0.28	0.65	0.58
*Tauraco hartlaubi*	Hartlaub’s turaco	0.27	0.30	0.83
*Chloropeta similis*	mountain yellow warbler	0.23	0.70	0.42
*Cyanomitra olivacea*	olive sunbird	0.13	0.12	0.25
*Psalidoprocne pristoptera*	black saw-wing	0.12	0.00	0.00
*Stephanoaetus coronatus*	crowned eagle	0.10	0.12	0.17
*Batis molitor*	chin-spot batis	0.10	0.00	0.00
*Parus albiventris*	white-bellied tit	0.08	0.00	0.33
*Pycnonotus barbatus*	common bulbul	0.08	0.00	0.25
*Pseudoalcippe abyssinica*	African hill babbler	0.07	0.00	0.42
*Phyllastrephus cabanisi*	Cabanis’s greenbul	0.07	0.05	0.17
*Schoutedenapus myoptilus*	scarce swift	0.07	0.00	0.08
*Muscicapa adusta*	African dusky flycatcher	0.07	0.12	0.75
*Chrysococcyx klaas*	Klaas’s cuckoo	0.07	0.00	0.08
*Streptopelia lugens*	dusky turtle dove	0.05	0.00	0.17

**Table 6.** Frequency of occurrence for species identified at the Dedan Kimathi University Wildlife Conservancy via point counts (PC), annotation of audio recordings (ARU) and citizen science (Kenya Bird Map, KBM).

species		survey method		
scientific name	common name	PC	ARU	KBM
*Pycnonotus barbatus*	common bulbul	0.70	0.62	0.15
*Camaroptera brachyura*	grey-backed camaroptera	0.68	0.80	0.33
*Cinnyris venustus*	variable sunbird	0.50	0.42	0.83
*Laniarius aethopicus*	tropical boubou	0.38	0.62	0.07
*Andropadus latirostris*	yellow-whiskered greenbul	0.28	0.80	0.37
*Dryoscopus cubla*	black-backed puffback	0.25	0.25	0.43
*Hedydipna collaris*	collared sunbird	0.23	0.55	0.43
*Melaenornis fischeri*	white-eyed slaty flycatcher	0.20	0.00	0.83
*Batis molitor*	chin-spot batis	0.15	0.45	0.00
*Apalis cinerea*	grey apalis	0.15	0.15	0.17
*Colius striatus*	speckled mousebird	0.12	0.10	0.91
*Pogoniulus bilineatus*	yellow-rumped tinkerbird	0.12	0.72	0.52
*Cossypha semirufa*	Rüppell’s robin chat	0.12	0.35	0.00
*Cossypha caffra*	Cape robin chat	0.12	0.30	0.00
*Psalidoprocne pristoptera*	black saw-wing	0.12	0.00	0.00
*Cisticola chiniana*	rattling cisticola	0.10	0.00	0.09
*Lanius collaris*	common fiscal	0.10	0.00	0.00
*Tauraco hartlaubi*	Hartlaub’s turaco	0.10	0.38	0.22
*Merops oreobates*	cinnamon-chested bee-eater	0.10	0.40	0.52
*Uraeginthus bengalus*	red-cheeked cordon-bleu	0.10	0.07	0.00

We examined our data in order to compare the frequency of occurrence of observed species between the PC data and ARU data for each site. We used the Kolmogorov–Smirnov test to determine if the differences in frequency of occurrence estimates were normally distributed and found that this was not the case at both sites (table 7). We therefore used the Wilcoxon signed-rank test to test the null hypothesis that there is no significant difference between the frequency of occurrence from PC data and ARU data.

**Table 7.** Kolmogorov–Smirnov test comparing the difference distribution to the standard normal. DeKUWC, Dedan Kimathi University Wildlife Conservancy; MKNP, Mount Kenya National Park.

site	statistic	p -value
DeKUWC	0.44	$4.3\times 10^{-20}$
MKNP	0.45	$3.6\times 10^{-12}$

There was no significant difference between the frequency of occurrence obtained from PC data and that from ARU data at MKNP (Wilcoxon signed-rank test: t=626.0; p=0.312, n=62 pairs), while at DeKUWC the frequency of occurrences between PC data and ARU data was significantly different (Wilcoxon signed-rank test: t=907.5; $p=4.23\times 10^{-6}$, n=110 pairs).

We repeated this statistical comparison between PCs and KBM and between KBM and ARUs. Table 8 shows a summary of the results obtained for both MKNP and DeKUWC. In each case, we show the p-value obtained by applying the Wilcoxon signed-rank test to test the null hypothesis that the two methods being compared are similar.

**Table 8.** Comparison of frequency of occurrence for different methods. DeKUWC, Dedan Kimathi University Wildlife Conservancy; MKNP, Mount Kenya National Park; PC, point counts; KBM, Kenya Bird Map; ARU, annotation of audio recordings.

site	methods	p -value
DeKUWC	PC–KBM	$4.93\times 10^{-28}$
	ARU–KBM	$1.49\times 10^{-17}$
MKNP	PC–KBM	$1.00\times 10^{-20}$
	ARU–KBM	$3.94\times 10^{-19}$

### (e) Machine learning performance

**Figure 8.**
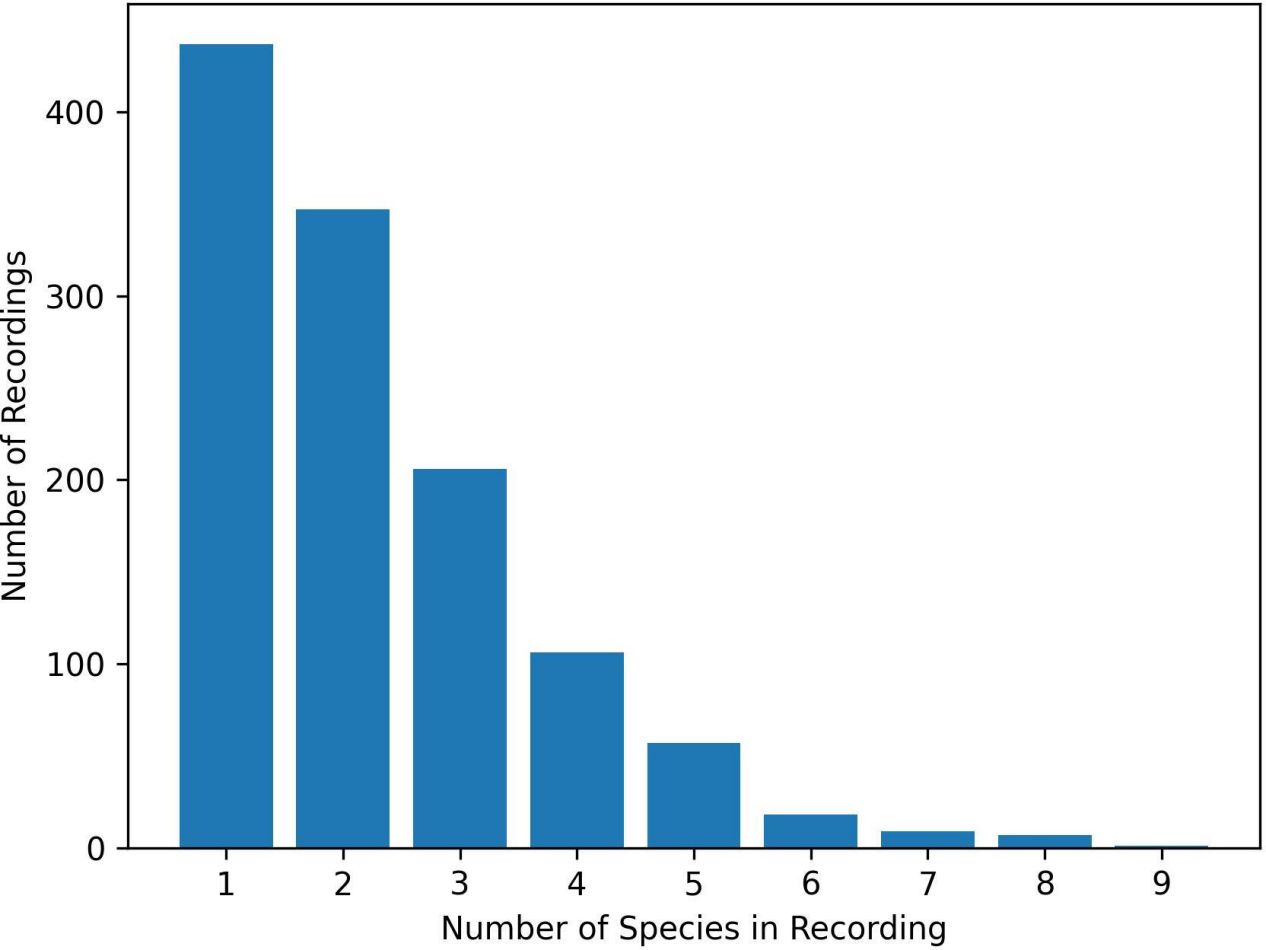
Number of species per recording in the 1188 annotated recordings containing at least one species.

Of the 1500 annotated audio recordings in our dataset, 1188 of them contain at least one species in either the foreground or background. Figure 8 shows the distribution of the number of species per recording. These recordings were used to test the performance of BirdNET and Perch and to train multi-label species recognition models using Perch embeddings. Table 9 shows the F1-Score for species that were present in at least 20 recordings for both BirdNET and Perch.

**Table 9.** Comparison of BirdNET, Perch and Finetuned MLP model on annotated recordings.

common name	number of recordings	BirdNET F1-score	Perch F1-score	MLP F1-score
brown woodland warbler	519	0.02	0.22	0.86
yellow-whiskered greenbul	221	0.56	0.68	0.69
grey-backed camaroptera	179	0.25	0.02	0.66
common bulbul	150	0.30	0.09	0.67
chestnut-throated apalis	132	0.00	0.00	0.42
yellow-rumped tinkerbird	124	0.43	0.49	0.58
Hartlaub's turaco	113	0.49	0.45	0.62
tropical boubou	113	0.36	0.33	0.49
red-fronted parrot	100	0.00	0.00	0.53
cinnamon bracken warbler	82	0.31	0.47	0.48
mountain yellow warbler	77	0.00	0.00	0.53
montane white-eye	76	0.00	0.00	0.23
white-starred robin	70	0.03	0.00	0.41
tambourine dove	50	0.30	0.29	0.55
collared sunbird	41	0.13	0.20	0.27
chin-spot batis	38	0.19	0.14	0.54
variable sunbird	38	0.05	0.05	0.25
black-backed puffback	33	0.31	0.39	0.24
olive thrush	31	0.00	0.00	0.12
Rüppell's robin chat	29	0.12	0.00	0.00
grey apalis	28	0.19	0.13	0.22
cinnamon-chested bee-eater	28	0.49	0.29	0.29
yellow-breasted apalis	22	0.06	0.06	0.00
Cape robin chat	22	0.24	0.36	0.18

A multi-label multilayer perceptron (MLP) was trained using the mean Perch embedding for each recording as the feature and the species present as the label. We focus on species present in at least 20 recordings and use a 70–30% train‐test split. Table 9 shows the F1-score performance for each species. We also performed 100 train‐test runs and obtained an average accuracy of 0.31 and an average macro-averaged area under the receiver operating characteristic (ROC) curve of 0.90.

## 4. Discussion

The 288 species identified in this study at both sites using PCs (94), identification in audio recordings by expert ornithologists (100) and KBM data (243) are within the range of the number of species known to occur in the region (e.g. Brooks T. *et al.* 1996*,* unpublished report), working in a similar area on the south western flank of Mount Kenya (Chehe and Ragati forests), managed to record only 102 bird species). It is also noteworthy that of these species, at DeKUWC, 177 were recorded by only one of the three methods, underscoring the value of having at least two methods in such initial surveys, especially combining KBM and ARUs. Our results are similar to those obtained elsewhere, e.g. [54], where the use of multiple survey methods is advocated. PCs registered the lowest number of unique species detected. However, PCs are designed for estimating absolute abundance [55] and long-term monitoring purposes [4].

At MKNP, the ARU did not detect as many unique species (5 at (figure 3) MKNP compared with 21 at DeKUWC (figure 2), probably reflecting a different avian community composition, which can influence method performance as much as habitat composition [56]. It is also likely that the 288 species recorded in the study were not entirely comprehensive, with previous work showing the complete bird species list for the larger Mount Kenya region at over 400 species [43].

From the species richness estimates obtained at both DeKUWC and MKNP, it can be seen that KBM records a much larger species richness than either PCs or ARUs. This can be explained by the relatively small number of samples required to achieve sampling completeness in citizen science projects such as KBM [57]. The KBM protocol mimics the timed species count method known for helping build checklists quickly [55,58]. There was also a much larger sampling effort represented by KBM and the greater diversity of microhabitats visited. When we focus on forest species, there is a difference in the two sites, with a greater number of forest specialists and generalists observed in MKNP than DeKUWC (figure 7). Though both sites are protected forests, MKNP is located far from human settlements. DeKUWC, on the other hand, is close to human settlements and has suffered from habitat degradation.

The observation of three species that are listed in the *IUCN red list of threatened species* is an indication that the study sites are important for the conservation of threatened species [53]. The MKNP is an important bird area[43], while the presence of the endemic and globally vulnerable Hinde’s babbler at DeKUWC would qualify the site as an Important Bird Area for hosting a globally threatened and restricted-range species [59]. However, no method picked up some globally threatened and/or rare species known to occur in Mount Kenya. Notable species from Mount Kenya that were not recorded include the elusive Abbott’s starling, for which Mount Kenya may be its stronghold, the African green ibis and the rare and little‐known race graueri of the African grass owl. This suggests that for rare or endemic species, the three methods we used may not be appropriate. Tailor-made species-specific approaches are recommended for such species [4].

The comparison of species’ relative abundance estimates obtained via PCs and ARUs presents mixed results in our study, with the two methods producing similar results for MKNP but differing for DeKUWC. For MKNP, as expected, we see that for species that are more heard than seen during PCs, 74% are also observed in the ARUs. We also see that the species that are more often heard than seen have high relative abundance as measured by their frequency of occurrence in both PCs and ARUs. In both sites, KBM results differ significantly from both PCs and ARUs. Hingston *et al.* [60] obtained similar results with differences between PCs and ARUs largely due to differences in detecting calls [60]. For example, during PCs, non-vocal species, which will be missed by ARUs, can be observed. In addition, some species are capable of mimicking calls of other species.

In comparing PCs and ARUs with KBM data, the species lists used for KBM were located within 20 km of the PC locations, and this could have introduced bias owing to changes in habitats surveyed. Using a reduced distance resulted in only one or two full protocol KBM lists. One advantage of citizen science is that we can scale monitoring efforts both temporally and spatially, e.g. [61,62]. However, as in the case of KBM at MKNP and DeKUWC, some important habitats may be inaccessible to citizen scientists or require significant effort to survey. The terrain in MKNP, for example, is difficult to traverse and consists of thick forest.

The performance of BirdNET and Perch for some Kenyan species is promising, with vocal species such as the yellow-whiskered greenbul, yellow-rumped tinkerbird and Hartlaub’s turaco having F1-scores of above 0.4 for both BirdNET and Perch (table 9). However, the performance of BirdNET and Perch reveals the need for fine-tuning these models with local data to improve performance. For example, for the brown woodland warbler, which is the most frequently occurring species in MKNP, for both PCs and ARU, BirdNET and Perch have F1-scores of 0.02 and 0.22, respectively. Performance improves to an F1-score of 0.86 in the model trained using Perch embeddings (table 9). This shows that it is possible to train machine learning models to recognize species in the Mount Kenya ecosystem using embeddings obtained from open-source pretrained models trained using much larger datasets when local recordings are available. In our case, a multi-label multi-layer perceptron obtained an average accuracy of 0.31 and an average macro-averaged area under the ROC curve of 0.90. This highlights the importance of collecting and annotating recordings from local ecosystems and making these recordings openly available for reuse. It also shows that there is a need to continue collection efforts in the Mount Kenya ecosystem to improve classifier accuracy.

The improved performance of machine learning models obtained by fine-tuning open source models in detecting Mount Kenya ecosystem species coupled with the result that in MKNP we observe no statistical difference in estimates of relative abundance of species via PCs and ARUs means that with improved machine learning models, monitoring comparable to PCs is achievable in some habitats using ARUs coupled with machine learning processing of the recordings. It also suggests that habitats can be monitored using vocal species such as the brown woodland warbler in MKNP for which training data can be readily obtained.

## 5. Conclusion

This article has presented a comparison of traditional PCs, the use of ARUs and citizen science to monitor bird species in the Mount Kenya ecosystem, with a focus on two sites. Our results reveal the need to combine multiple approaches when conducting bird surveys, with each technique having advantages and disadvantages. In addition, the performance of different methods varies with habitat. We note that citizen science is useful for building species lists quickly, but with few records in areas that are difficult to access. The KBM protocol mimics timed species counts that give observers the flexibility to investigate all the microhabitats in an area and is therefore useful for building species lists quickly, while ARUs are useful for picking up skulking species that are more often heard than seen. This makes the combination of methods ideal, especially now that policy-makers and conservation practitioners are using biodiversity indices to gauge conservation success and failure [63,64]. In addition, the integration of machine learning models into data processing pipelines is a promising avenue for future work, with the results obtained here showing that relatively good results can be obtained for vocal species by leveraging open-source pretrained models.

## Data Availability

Data associated with this paper are available on Dryad at [40]. Code to reproduce the analysis is on Zenodo at [51] and the accompanying GitHub repository: https://github.com/DeKUT-DSAIL/ndege-zetu.
